# The power metric: a new statistically robust enrichment-type metric for virtual screening applications with early recovery capability

**DOI:** 10.1186/s13321-016-0189-4

**Published:** 2017-02-02

**Authors:** Julio Cesar Dias Lopes, Fábio Mendes dos Santos, Andrelly Martins-José, Koen Augustyns, Hans De Winter

**Affiliations:** 1 NEQUIM - Chemoinformatics Group, Departamento de Quimica, Universidade Federal de Minas Gerais, Belo Horizonte, Brazil; 2 Medicinal Chemistry Group, Department of Pharmaceutical Sciences, University of Antwerp, Campus Drie Eiken, Building A, Universiteitsplein 1, 2610 Wilrijk, Antwerp Belgium

**Keywords:** Power metric (PM), Virtual screening, Metric, Model performance, Enrichment factor, Area under the curve (AUC), Receiver operating curve enrichment factor (ROCE), Correct classification rate (CCR), Matthews correlation coefficient (MCC), Cohen’s kappa coefficient (CKC), Relative enrichment factor (REF)

## Abstract

A new metric for the evaluation of model performance in the field of virtual screening and quantitative structure–activity relationship applications is described. This metric has been termed the power metric and is defined as the fraction of the true positive rate divided by the sum of the true positive and false positive rates, for a given cutoff threshold. The performance of this metric is compared with alternative metrics such as the enrichment factor, the relative enrichment factor, the receiver operating curve enrichment factor, the correct classification rate, Matthews correlation coefficient and Cohen’s kappa coefficient. The performance of this new metric is found to be quite robust with respect to variations in the applied cutoff threshold and ratio of the number of active compounds to the total number of compounds, and at the same time being sensitive to variations in model quality. It possesses the correct characteristics for its application in early-recognition virtual screening problems.

## Background

The field of virtual screening with applications in drug design has become increasingly important in terms of hit finding and lead generation [1–3]. Many different methods and descriptors have emerged over time to help the drug discovery scientist in applying the most optimal techniques for almost any given computational problem [4]. However, still a serious drawback in the domain of virtual screening is the lack of metrics standards to statistically evaluate and compare the performance of different methods and descriptors. Nicholls [5] suggested a few list of desirable characteristics of a good metric:

1. independence to extensive variables,
2. statistical robustness,
3. straightforward assessment of error bounds,
4. no free parameters,
5. easily understandable and interpretable.

In addition to these five characteristics, we believe that a good metric might also benefit from having well-defined lower and upper boundaries as this facilitates quantitative comparison of different models and facilitates optimization of fitness functions based on these metrics.

In this paper a new metric is proposed that adheres to the six desired characteristics of an ideal metric. The metric is based on the principles behind the power of hypothesis test, which is the probability of making the correct decision if the alternative hypothesis is true. Comparison of the new power metric with more established metrics, including the enrichment factor (EF) [6, 7], the relative enrichment factor (REF) [8], the receiver operating characteristic (ROC) enrichment ROCE [9–11], the correct classification rate (CCR) [12, 13], Matthews correlation coefficient (MCC) [14], Cohen’s kappa coefficient (CKC) [15, 16] together with the standard precision (PRE), accuracy (ACC), sensitivity (SEN) and specificity (SPE) metrics, is presented in this paper.

## Methods

### Definitions

In the field of virtual screening, the quality of a model can be quantified by a number of metrics. The area under the curve (AUC) represents the overall accuracy of a model, with a value approaching 1.0 indicating a high sensitivity and high specificity [17]. A model with an AUC of 0.5 represents a test with zero discrimination. AUC metrics are calculated from typical ROC curves; these are plots of the (1 − SPE) values on the *x*-axis against the SEN values plotted on the *y*-axis for all possible cutoff points. Sensitivity and specificity, and thus the AUC, are good indicators of the validity of a method but are not measuring the predictive value of a method [18].

**Fig. 1:**
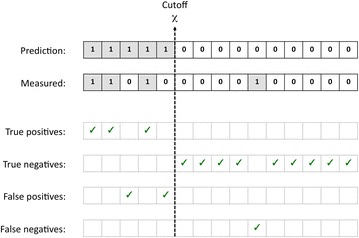
Illustration of the relation between cutoff *χ* and number of predicted actives/non-actives. Assuming a list of compounds ranked according to their predicted activity values, all compounds that are located on the *left side* of *χ* on this ranked list are predicted to be active, while all compounds that are located on the *right-hand side* of *χ* on this list are predicted to be non-active. All compounds that fall along the *left-hand side* of *χ* define the ‘selection set’; in this example this includes five compounds. The total number of compounds in the selection set is *N*_*s*_ (here: 5), while the total number of compounds in the entire collection is *N* (here: 15). The number of true actives in the selection set is *n*_*s*_ (here: 3) and the number of true actives in the entire data collection is *n* (here: 4). Using these abbreviations, one can define the number of true positives TP as being equal to *n*_*s*_, the number of true negatives TN equal to (*N* − *N*_*s*_ − *n* + *n*_*s*_), the number of false positives FP equal to (*N*_*s*_ − *n*_*s*_), and the number of false negatives FN being equal to (*n* − *n*_*s*_)

The AUC is a metric that describes the overall quality of a model. In practical virtual screening experiments however, it is typical to score each molecule according to a value proposed by the model, and rank these molecules in decreasing order based on these calculated values. It is custom to define a cutoff threshold *χ* that separates predicted actives (all compounds along the ‘top’ side of this ranked list) from predicted non-actives (all compounds along the ‘bottom’ side of the ranked list) (see Fig. 1). The cutoff threshold *χ* is defined as the fraction of compounds selected:

1 $$\chi = N_{s} /N$$

with *N*_*s*_ being the number of compounds in the selection set (the predicted actives) and *N* being the total number of compounds in the entire dataset. The majority of metrics, including all metrics in this paper, are dependent on the value of this cutoff criterion *χ* since this criterion defines which compounds are predicted to be active and non-active.

Apart from the *N*_*s*_ and *N* variables, two other definitions are used in the following sections: the number of true active compounds in the selection set that is defined as *n*_*s*_, and the number of true active compounds in the entire dataset defined as *n*. Finally, the prevalence of actives *R*_*a*_ in the entire dataset can be defined as:

2 $$R_{a} = n/N$$

### Definition and calculation of established metrics

The sensitivity of a model is defined as the ability of the model to correctly identify active compounds from all the actives in the screening set (also termed the true positive rate or TPR), while specificity refers to the ability of the model to correctly identify inactives from all inactives in the dataset at a given cutoff threshold χ:

3 $${SEN}\left( \chi \right) = TPR\left( \chi \right) = \frac{TP}{TP + FN} = \frac{{n_{s} }}{n}$$

4 $$SPE\left( \chi \right) = \frac{TN}{FP + TN} = \frac{{N - N_{s} - n + n_{s} }}{N - n}$$

In line with the true positive rate, one can also define a false positive rate FPR as the number of true inactives in the selection set in relation to the total number of inactives in the entire dataset:

5 $$FPR\left( \chi \right) = \frac{FP}{FP + TN} = \frac{{N_{s} - n_{s} }}{N - n}$$

Other well-established metrics include the precision and accuracy:

6 $$PRE\left( \chi \right) = \frac{TP}{TP + FP} = \frac{{n_{s} }}{{N_{s} }}$$

7 $$ACC\left( \chi \right) = \frac{TP + TN}{TP + TN + FP + FN} = \frac{{2n_{s} + N - N_{s} - n}}{N}$$

The enrichment factor is probably the most used metric in virtual screening and other fields as well. The EF at a given cutoff *χ* is calculated from the proportion of true active compounds in the selection set in relation to the proportion of true active compounds in the entire dataset:

8 $$EF\left( \chi \right) = \frac{TP/TP + FP}{TP + FN/TP + TN + FP + FN} = \frac{{N \times n_{s} }}{{n \times N_{s} }}$$

The enrichment factor is very intuitive and easy to understand, but it lacks a strong statistic background and has some drawbacks, including the lack of a well-defined upper boundary [the EF(χ) can vary from 0 in the case that there are no active compounds in the selection set (*n*_*s*_ = 0), and up to 1/*χ* when all active compounds are located in the selection set (*n*_*s*_ = *n*); see Ref. [19] for the derivation], the dependency of the value on the ratio of active to inactive compounds in the dataset, and a pronounced ‘saturation effect’ when the actives saturate the early positions of the ranking list and the performance metric cannot get any higher, thereby preventing to distinguish between good and excellent models [6].

To avoid the problems associated to EF, a number of other metrics have been proposed. The first of these is the relative enrichment factor [8], a metric in which the problem associated to the saturation effect is fixed by considering the maximum EF achievable at the cutoff point:

9 $$REF\left( \chi \right) = \frac{{100 \times n_{s} }}{{\hbox{min} \left( {N \times \chi ,n} \right)}}$$

The REF, has well defined boundaries—ranging from 0 to 100—and is less subject to the saturation effect.

The ROC enrichment metric is defined as the fraction of actives found when a given fraction of inactives has been found [9]:

10 $$\it {{ROCE}}\left(\upchi \right) = \frac{{{{n}}_{{s}} /{{n}}}}{{\left( {{{N}}_{{s}} - {{n}}_{{s}} } \right)/\left( {{{N}} - {{n}}} \right)}} = \frac{{{{n}}_{{s}} \times \left( {{{N}} - {{n}}} \right)}}{{{{n}} \times \left( {{{N}}_{{s}} - {{n}}_{{s}} } \right)}}$$

The ROCE metric has been advocated by some researches as a better approach to address early recovery [5, 9]. However, some issues still remain, such as the lack of a well-defined upper boundary [which is equal to 1/*χ* when TPR*(χ)* equals 1], a smaller but still noticeable saturation effect, and a statistic robustness which is not as desirable as we will demonstrate later.

Another metric often considered to measure classification performances is the correct classification rate [12], defined as the percentage of instances correctly classified:

11 $$\it {{CCR}}\left(\upchi \right) = \frac{1}{2}\left[ {\frac{{TP}}{{{{TP}} + {{FN}}}} + \frac{{TN}}{{{{TN}} + {{FP}}}}} \right] = \frac{1}{2}\left[ {\frac{{{{n}}_{{s}} }}{{n}} + \frac{{{{N}} - {{N}}_{{s}} - {{n}} + {{n}}_{{s}} }}{{{{N}} - {{n}}}}} \right]$$

The CCR is sometimes also called the balanced accuracy [20].

Matthews correlation coefficient has been advocated as a balanced measure that can be used on classes of different sizes [14]. The MCC is in essence a correlation coefficient between the measured and predicted classifications; it returns a coefficient of +1 in the case of a perfect prediction, 0 when no better than random prediction and −1 in cases of total disagreement between prediction and observation:

12 $$\it {{MCC}}\left(\upchi \right) = \frac{TP \times TN - FP \times FN}{{\sqrt {\left( {TP + FP} \right)\left( {TP + FN} \right)\left( {TN + FP} \right)\left( {TN + FN} \right)} }} = \frac{{N \times n_{s} - N_{s} \times n}}{{\sqrt {N_{s} \times n \times \left( {N - n} \right) \times \left( {N - N_{s} } \right)} }}$$

The last metric that is evaluated with respect to its performance as compared to the here developed power metric is Cohen’s kappa coefficient [21–24]:

13 $$\it {{CKC}}\left(\upchi \right) = 1 - \frac{{1 - \frac{TP + TN}{TP + TN + FP + FN}}}{{1 - \frac{{\left( {TP + FN} \right)\left( {TP + FP} \right) + \left( {FP + TN} \right)\left( {FN + TN} \right)}}{{\left( {TP + TN + FP + {FN}^{2}} \right) }}}} = 1 - \frac{{N \times n + N \times N_{s} - 2 \times n_{s} \times N}}{{N \times n + N \times N_{s} - 2 \times n \times N_{s} }}$$

### Derivation of a new metric: the power metric

In virtual screening studies, we can assume all compounds being inactive as the null hypothesis, and the assumption that some compounds are active as the alternative hypothesis. The statistical power, also known as sensitivity or recall, is equal to the true positive rate.

However, the statistical power alone does not include information about the distribution of negative instances or the size effect. Therefore, a metric based on statistical power and suited for applications in the field of virtual screening should incorporate information about the negative instances as well. Ideally, a good virtual screening method must be able to perform a good prediction of true positive instances combined with a small false positive prediction rate. This translates in a metric that combines the TPR with the false positive rate:

14 $$'net\;power '\;(\chi ) = TPR(\chi ) - FPR(\chi )$$

**Fig. 2:**
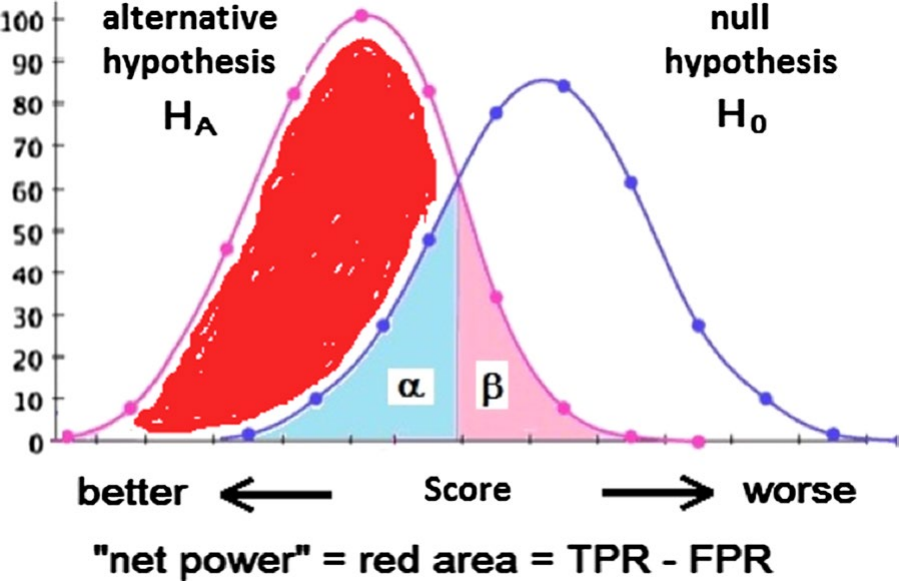
Distribution curves of null and alternative hypothesis. The *red area* is the area defined by the alternative hypothesis minus the area defined by the null hypothesis, or, put differently, as the true positive rate (TPR, or 1 − *β*) minus the false positive rate (FPR, or *α* or type I error), hence ‘net power’ = 1 – *β* − *α*. The cutoff point is defined as the crossing point of the two distributions

Graphically, the ‘net power’ is the area of the distribution of positive instances or the alternative hypothesis, minus the area of the distribution of negative instances or the null hypothesis (Fig. 2).

The metric is not new; it has been developed independently several times in the past. Its origin can be traced back to the seminal paper of Peirce [25] with his ‘science of the method’ [26]. More than 70 years later, it was proposed again by Youden as Youden’s index (*Y’J*) [27]. Youden’s index is often used in conjunction with the ROC curve as a criterion for selecting the optimum cutoff point [28]. The index has been used to calculate the best cutoff point in the ROC curve. Once more, almost 50 years later in 2003, it was proposed again by Powers who called it ‘informedness’ [10].

Despite the success of this metric to evaluate the prediction power of a method, it is not entirely appropriate for virtual screening studies due to the lack of early recovery capabilities that are very desirable in any virtual screening application. Consider, for instance, a database of 10,000 compounds of which 1% are active compounds. In this hypothetical thought experiment, we can think of different methods that yield identical Youden’s indices calculated from different TPR and FPR values. Thinking of two methods, each produce a Youden’s index of 0.5, with the first one characterized by a TPR = 0.9 and a FPR = 0.4, and the second method characterized by a TPR = 0.51 and a FPR = 0.01. In the case of the first method, 4050 compounds will be marked as ‘hits’ of which only 90 compounds being true active (or 5.7% of the selected compounds). However, for the second method only 150 compounds are flagged as ‘hits’, of which 51 compounds are true actives (or 34% of the selected compounds). Obviously, for virtual screening applications, the second method provides a more optimal early recovery rate since only 1.5% of the original dataset needs to be tested in order to recover 51% of all active compounds.

Normalization of the ‘net power’ metric by dividing by the sum of the true positive and false negative rates introduces early recovery capabilities bias into the ‘net power’ metric. This difference-over-the-sum normalized ‘net power’ expresses the dominance of the true positive rate over the false positive rate among those instances predict as positive, expressed by its rates:

15 $$normalized\;'net power^{\prime} = \frac{TPR\left( \chi \right) - FPR\left( \chi \right)}{TPR\left( \chi \right) + FPR\left( \chi \right)}$$

The metric ranges from −1 to +1 and can easily be modified to range from 0 to +1 by adding 1 to the metric and dividing by 2. We call this new metric the power metric (PM) and is defined as follows:

16 $${PM}\left( \chi \right) = \frac{{\left( {\frac{TPR\left( \chi \right) - FPR\left( \chi \right)}{TPR\left( \chi \right) + FPR\left( \chi \right)} + 1} \right)}}{2} = \frac{TPR\left( \chi \right)}{TPR\left( \chi \right) + FPR\left( \chi \right)} = \frac{{n_{s} \times N - n \times n_{s} }}{{n_{s} \times N - 2 \times n \times n_{s} + n \times N_{s} }}$$

### Probability distribution function to evaluate the metrics

In order to evaluate the performance of several metrics used in the field of virtual screening, we used the probability distribution function approach as suggested by Truchon and Bayly to build hypothetical models of different qualities [6]. For a typical virtual screening study with *N* compounds of which *n* being active compounds, we generated the ranks of these active compounds according to the exponential distribution as proposed by Truchon and Bayly [6]:

17 $$X_{i} = \frac{ - 1}{\lambda }ln\left( {1 - U_{i} \left( {1 - e^{ - \lambda } } \right)} \right)$$

The generated real number *X*_*i*_ corresponds to the relative position of active compound *i* and *U*_*i*_ is a pseudo random number with values between 0 and 1. In this exponential distribution, the *λ* parameter represents the model quality (lower *λ* values correspond to poor models and larger *λ* values correspond to better models). The number *X*_*i*_ is transformed into a rank integer *r*_*i*_ that falls within 1 and *N*:

18 $$r_{i} = int\left( {N \times X_{i} + 0.5} \right)$$

**Fig. 3:**
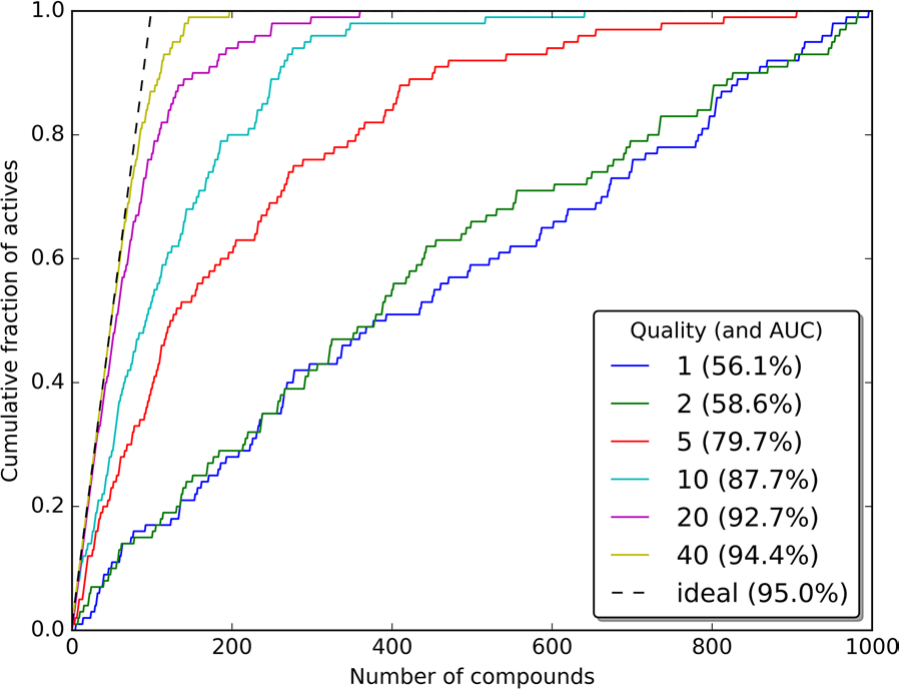
Visualization of the quality of the test models. In each case, 100 active compounds were distributed in a total set of 1000 compounds according to the distribution as defined by Eq. 17. The term ‘quality’ corresponds to the *λ* value of Eq. 17. The ‘ideal’ case was generated by positioning all 100 actives at the top-100 positions of the dataset. For each model, the AUC was calculated by integration using the composite trapezoidal rule

No ties were allowed and each active compound occupies one unique position. In cases when a clash occurred, a new random number was generated. In our simulations we used values of *λ* equal to 1, 2, 5, 10, 20 and 40. Visualization of the quality of these models is given in Fig. 3.

To illustrate the model generation process by example, consider a model with quality *λ* = 20 and consisting of *n* = 100 active compounds on a total of *N* = 10,000 compounds. To generate the relative rankings of these 100 active compounds, Eq. 17 was called 100 times, each time with a different random number *U*_*i*_. Using Eq. 18, the 100 generated *X*_*i*_ numbers are then converted into 100 rankings *r*_*i*_ with *N* set to 10,000. These 100 rankings are the absolute positions of the active compounds; the remaining 9900 ranks (10,000 − 100 = 9900) are those of the inactive compounds.

In order to evaluate the quality of the PM metric and to compare its behavior to the other metrics, a large number of datasets were generated and analyzed. The total number of compounds *N*, number of actives *n*, model quality *λ* and cutoff parameter *χ* were varied. Each simulation was repeated 10,000 times and the results were analyzed by inspecting the variations of mean and standard deviation (STD) of the metrics as a function of the number of actives and total compounds. The eleven enrichment-type metrics that were analyzed were the PM, EF, ROCE, CCR, REF, MCC, CKC, together with the standard PRE, ACC, SEN and SPE metrics.

All calculations were performed under Python 2.7 using Numpy and Scipy [29]. The IPython notebook [30] was used as programming environment and figures were generated with Matplotlib [31]. MarvinSketch was used for drawing chemical structures [32].

## Results and discussion

### Dependency on model quality

One of the key aspects of a suitable metric is that its value is dependent of the model quality. In Table 1, the dependency of the different metrics on the model quality parameter *λ* was evaluated. All metrics are model quality dependent, but the ROCE, EF, REF, MCC, CKC, SEN and PRE show an approximate tenfold increase when moving from a poor model with quality *λ* = 2 to a good model with quality *λ* = 40, while in the case of the PM metric a doubling of the parameter value is observed (going from PM = 0.5 for a poor model to a value of 0.98 for a good model; Table 1). Accuracy and specificity metrics are not influenced by the model quality *λ* or by the cutoff value *χ*; both metrics fluctuate around a value of 0.97-1.00 irrespective of the underlying model quality or applied threshold cutoff. In the case of the CCR metric, the maximal value of this metric finds it limit at 0.75 ± 0.02 for the case with an extremely good model quality of *λ* = 40 in combination with a threshold cutoff *χ* of 2% (for a model with 100 actives on a total of 10,000 compounds, a model quality of *λ* = 40 corresponds to an AUC of 97.25%, as compared to an AUC of 99.5% for the ideal case). This is not what one would like to expect for a metric to separate quality models from poor models. Furthermore, the PM metric seems to be less influenced by the applied cutoff parameter *χ*, since the PM metric for a good model (*λ* = 40) at the different cutoffs of 0.5, 1 and 2% remains largely unchanged (at a constant value of approximately 0.98; see Table 1), while an increase is seen for the CCR metric. It seems that all but the PM, SPE and ACC metrics are more dependent on the applied cutoff threshold *χ* (indicated by the shifts in the values and by the larger variations on the calculated metrics; Table 1), making it more difficult to define an appropriate metric value for identification proper virtual screening models. Starting with models of reasonable quality, and up to models of higher qualities (*λ* ≥ 10), the PM is calculated to vary between 0.9 and 1.0 with a relative standard deviation less than 10%. For the other metrics (except the CCR, ACC and SPE metrics), this relative standard deviation is in most instances larger than 10%.

**Table 1:** Dependency on the model quality parameter *λ* using models generated from datasets with 100 actives (*n*) on 10,000 compounds in total (*N*)

Metric	*λ*					*χ* (%)
	2	5	10	20	40	
PM	0.51 ± 0.35	0.74 ± 0.24	0.89 ± 0.09	0.95 ± 0.02	0.98 ± 0.01	0.5
ROCE	2.35 ± 2.18	5.13 ± 3.39	10.46 ± 4.99	22.34 ± 7.86	49.96 ± 14.35	
EF	2.28 ± 2.06	4.83 ± 3.03	9.38 ± 4.04	18.08 ± 5.17	32.94 ± 6.22	
REF	2.28 ± 2.06	4.83 ± 3.03	9.38 ± 4.04	18.08 ± 5.17	32.94 ± 6.22	
CCR	0.50 ± 0.01	0.51 ± 0.01	0.52 ± 0.01	0.54 ± 0.01	0.58 ± 0.02	
MCC	0.01 ± 0.01	0.03 ± 0.02	0.06 ± 0.03	0.12 ± 0.04	0.23 ± 0.04	
CKC	0.01 ± 0.01	0.03 ± 0.02	0.06 ± 0.03	0.11 ± 0.03	0.21 ± 0.04	
SEN	0.01 ± 0.01	0.02 ± 0.02	0.05 ± 0.02	0.09 ± 0.03	0.16 ± 0.03	
SPE	1.00 ± 0.00	1.00 ± 0.00	1.00 ± 0.00	1.00 ± 0.00	1.00 ± 0.00	
PRE	0.02 ± 0.02	0.05 ± 0.03	0.09 ± 0.04	0.18 ± 0.05	0.33 ± 0.06	
ACC	0.99 ± 0.00	0.99 ± 0.00	0.99 ± 0.00	0.99 ± 0.00	0.99 ± 0.00	
PM	0.61 ± 0.23	0.80 ± 0.11	0.90 ± 0.04	0.95 ± 0.01	0.98 ± 0.00	1
ROCE	2.32 ± 1.55	5.07 ± 2.31	10.19 ± 3.34	20.97 ± 5.08	44.00 ± 8.22	
EF	2.26 ± 1.48	4.83 ± 2.09	9.25 ± 2.75	17.33 ± 3.46	30.54 ± 3.95	
REF	2.26 ± 1.48	4.83 ± 2.09	9.25 ± 2.75	17.33 ± 3.46	30.54 ± 3.95	
CCR	0.51 ± 0.01	0.52 ± 0.01	0.54 ± 0.01	0.58 ± 0.02	0.65 ± 0.02	
MCC	0.01 ± 0.02	0.04 ± 0.02	0.08 ± 0.03	0.17 ± 0.03	0.30 ± 0.04	
CKC	0.01 ± 0.02	0.04 ± 0.02	0.08 ± 0.03	0.17 ± 0.03	0.30 ± 0.04	
SEN	0.02 ± 0.01	0.05 ± 0.02	0.09 ± 0.03	0.17 ± 0.03	0.31 ± 0.04	
SPE	0.99 ± 0.00	0.99 ± 0.00	0.99 ± 0.00	0.99 ± 0.00	0.99 ± 0.00	
PRE	0.02 ± 0.01	0.05 ± 0.02	0.09 ± 0.03	0.17 ± 0.03	0.31 ± 0.04	
ACC	0.98 ± 0.00	0.98 ± 0.00	0.98 ± 0.00	0.98 ± 0.00	0.99 ± 0.00	
PM	0.66 ± 0.13	0.82 ± 0.06	0.90 ± 0.02	0.95 ± 0.01	0.97 ± 0.00	2
ROCE	2.30 ± 1.08	4.91 ± 1.56	9.69 ± 2.18	18.75 ± 3.08	35.21 ± 4.06	
EF	2.26 ± 1.03	4.70 ± 1.43	8.88 ± 1.82	15.87 ± 2.19	26.17 ± 2.23	
REF	4.52 ± 2.07	9.40 ± 2.85	17.76 ± 3.65	31.74 ± 4.38	52.34 ± 4.45	
CCR	0.51 ± 0.01	0.54 ± 0.01	0.58 ± 0.02	0.65 ± 0.02	0.75 ± 0.02	
MCC	0.02 ± 0.01	0.05 ± 0.02	0.11 ± 0.03	0.21 ± 0.03	0.36 ± 0.03	
CKC	0.02 ± 0.01	0.05 ± 0.02	0.11 ± 0.02	0.20 ± 0.03	0.34 ± 0.03	
SEN	0.05 ± 0.02	0.09 ± 0.03	0.18 ± 0.04	0.32 ± 0.04	0.52 ± 0.04	
SPE	0.98 ± 0.00	0.98 ± 0.00	0.98 ± 0.00	0.98 ± 0.00	0.99 ± 0.00	
PRE	0.02 ± 0.01	0.05 ± 0.01	0.09 ± 0.02	0.16 ± 0.02	0.26 ± 0.02	
ACC	0.97 ± 0.00	0.97 ± 0.00	0.97 ± 0.00	0.98 ± 0.00	0.98 ± 0.00	

Metric abbreviations are given in the Methods section. All metrics are dependent on the model quality, but in case of the ROCE, EF, REF, MCC, CKC, SEN and PRE metrics there is at least a tenfold increase when moving from a bad model (*λ* = 2) to a good model (*λ* = 40), while for the PM metric there is a doubling of the value. The accuracy ACC and specificity SPE metrics are not dependent on the quality of model, while the correct classification rate metric (CCR) shifts from 0.5 in the case of a bad model to a maximum of 0.75 for the best model. Good models have a PM of >0.9; for good models this value is largely independent on the applied cutoff value *χ* (see Table 3 as well)

### Dependency on the ratio of actives to total number of compounds

The influence of the *R*_*a*_ value, calculated from the ratio of number of actives *n* to the total number of compounds *N*, on the different metrics is given in Table 2. For the different model qualities (a poor model with *λ* = 1 or a good model with *λ* = 20) and different cutoff values (*χ* = 1 or 10%), there is a significant dependency for the REF, PRE and ACC metrics on the *R*_*a*_ value. The EF, CKC, SEN and ROCE metrics are not very sensitive to the *R*_*a*_ value when applied to poor models (*λ* = 1), but show more dependency on the *R*_*a*_ ratio when applied on good models (*λ* = 20). In contrast, the REF is very sensitive to the *R*_*a*_ value when used on poor models (*λ* = 1), but is not dependent on the *R*_*a*_ value when applied on a good model in combination with a large cutoff value (*χ* = 1%; Table 2). In contrast, the PM and CCR metrics remain largely insensitive to the *R*_*a*_ value, unless when the PM metric it is applied to a very poor model (*λ* = 1) in combination with a small cutoff threshold value (*χ* = 1%). Again, good models all have PM values ≥ 0.9 with small variations, and are independent on the number of actives in relation to the total number of compounds. The combination of a high model quality of *λ* = 20 with a cutoff threshold of *χ* = 1%, applied to a database with *n* = 50 actives on a total of *N* = 5000 compounds, corresponds to a virtual screening situation characterized by a high true positive and high true negative rate. It is therefore surprising that for the CCR metric a value of 0.58 ± 0.02 is calculated, while for the PM metric a more intuitive value of 0.95 ± 0.02 is found (Table 2). Increasing the cutoff threshold to 10% improves the calculated CCR value to 0.88 ± 0.02 and decreases the PM case from 0.95 ± 0.02 to 0.90 ± 0.01, again in line what one would expect from considering the true positive and true negative rates in this situation.

**Table 2:** Dependency on the *R*_*a*_ value

Metric	*R*_*a*_			*χ* (%)	*λ*
	0.01 (*n* = 50; *N* = 5000)	0.05 (*n* = 250; *N* = 5000)	0.2 (*n* = 1000; *N* = 5000)		
PM	0.39 ± 0.36	0.57 ± 0.15	0.62 ± 0.07	1	1
ROCE	1.59 ± 1.83	1.62 ± 0.85	1.73 ± 0.54		
EF	1.55 ± 1.75	1.54 ± 0.74	1.48 ± 0.32		
REF	1.55 ± 1.75	7.69 ± 3.71	29.58 ± 6.38		
CCR	0.50 ± 0.01	0.50 ± 0.00	0.50 ± 0.00		
MCC	0.01 ± 0.02	0.01 ± 0.02	0.02 ± 0.02		
CKC	0.01 ± 0.02	0.01 ± 0.01	0.01 ± 0.01		
SEN	0.02 ± 0.02	0.02 ± 0.01	0.01 ± 0.00		
SPE	0.99 ± 0.00	0.99 ± 0.00	0.99 ± 0.00		
PRE	0.02 ± 0.02	0.08 ± 0.04	0.30 ± 0.06		
ACC	0.98 ± 0.00	0.94 ± 0.00	0.80 ± 0.00		
PM	0.58 ± 0.09	0.60 ± 0.04	0.62 ± 0.02	10	
ROCE	1.50 ± 0.51	1.53 ± 0.24	1.62 ± 0.15		
EF	1.49 ± 0.49	1.49 ± 0.22	1.44 ± 0.09		
REF	14.88 ± 4.95	14.88 ± 2.16	28.73 ± 1.87		
CCR	0.52 ± 0.03	0.53 ± 0.01	0.53 ± 0.01		
MCC	0.02 ± 0.02	0.04 ± 0.02	0.07 ± 0.02		
CKC	0.01 ± 0.01	0.03 ± 0.02	0.07 ± 0.01		
SEN	0.15 ± 0.05	0.15 ± 0.02	0.14 ± 0.01		
SPE	0.90 ± 0.00	0.90 ± 0.00	0.91 ± 0.00		
PRE	0.01 ± 0.00	0.07 ± 0.01	0.29 ± 0.02		
ACC	0.89 ± 0.00	0.86 ± 0.00	0.76 ± 0.00		
PM	0.95 ± 0.02	0.98 ± 0.01	1.00 ± 0.00	1	20
ROCE	21.06 ± 7.29	46.82 ± 15.58	nan^a^		
EF	17.24 ± 4.92	13.94 ± 1.27	5.00 ± 0.00		
REF	17.24 ± 4.92	69.71 ± 6.35	100.00 ± 0.00		
CCR	0.58 ± 0.02	0.57 ± 0.01	0.53 ± 0.00		
MCC	0.16 ± 0.05	0.30 ± 0.03	0.20 ± 0.00		
CKC	0.16 ± 0.05	0.22 ± 0.02	0.08 ± 0.00		
SEN	0.17 ± 0.05	0.14 ± 0.01	0.05 ± 0.00		
SPE	0.99 ± 0.00	1.00 ± 0.00	1.00 ± 0.00		
PRE	0.17 ± 0.05	0.70 ± 0.06	1.00 ± 0.00		
ACC	0.98 ± 0.00	0.95 ± 0.00	0.81 ± 0.00		
PM	0.90 ± 0.01	0.93 ± 0.00	1.00 ± 0.00	10	
ROCE	9.30 ± 0.57	13.38 ± 0.59	1612.74 ± 529.71		
EF	8.58 ± 0.49	8.26 ± 0.22	4.99 ± 0.01		
REF	85.82 ± 4.86	82.60 ± 2.15	99.84 ± 0.18		
CCR	0.88 ± 0.02	0.88 ± 0.01	0.75 ± 0.00		
MCC	0.25 ± 0.02	0.56 ± 0.02	0.67 ± 0.00		
CKC	0.14 ± 0.01	0.52 ± 0.02	0.61 ± 0.00		
SEN	0.86 ± 0.05	0.83 ± 0.02	0.50 ± 0.00		
SPE	0.91 ± 0.00	0.94 ± 0.00	1.00 ± 0.00		
PRE	0.09 ± 0.00	0.41 ± 0.01	1.00 ± 0.00		
ACC	0.91 ± 0.00	0.93 ± 0.00	0.90 ± 0.00		

In the case of bad model quality (*λ* = 1), the metrics most sensitive to variations in the *R*_*a*_ value include the REF, PRE and ACC metrics, and also the CKC metric in the case of a large cutoff value of *χ* = 10%. This dependency is not so outspoken for the PM metric, except in the case when a very bad model is combined with a low cutoff value (*χ* = 1%). In cases with better model quality (*λ* = 20), significant dependencies are observed for the ROCE, EF, REF, MCC, CKC, SEN, PRE and ACC metrics, while the PM, CCR and SPE metrics are more stable. The metric that is least sensitive to variations in the *R*_*a*_ value, irrespective of the underlying model quality or cutoff threshold, is the CCR metric

^a^In this case the ROCE metric could not be calculated from Eq. 10 since (*N*_*s*_ − *n*_*s*_) is equal to 0

### Dependency on the cutoff threshold χ

The dependency of the different metrics on the applied cutoff value *χ* is given in Table 3. This dependency was evaluated using models with *n* = 250 active compounds in a dataset of *N* = 10,000 compounds in total, and at five different cutoff values *χ* (0.5, 1, 2.5, 5 and 10%) for both a poor and high quality model (*λ* = 1 and 20, respectively). A significant dependency on the cutoff *χ* is observed for the REF and SEN metrics, increasing their values with increasing cutoff values. A similar behavior is observed for the CCR, MCC and CKC metrics when applied to the high quality model situation (*λ* = 20). Interestingly, the calculated REF metric values remain constant up to a cutoff of 2.5%, but at higher cutoff values this metric increases significantly. It is not surprising that this turning point in metric behavior is observed at a cutoff value of 2.5%, since this corresponds to a selection set of exactly 250 compounds when applied to a dataset of 10,000 compounds with 250 actives mixed into it. In case of a high quality model, this translates to a situation with maximum rates of true positives and true negatives. Focusing on the EF, ROCE, CCR, SPE, ACC and PM metrics, their values are quite constant over the different cutoff values in the case of a bad model quality, but a significant drift is observed for the EF, CCR and ROCE metrics in case of a good model quality. This shift is again observed at a *χ* cutoff value larger than 2.5%. A similar drift is not observed for the PM metric that, together with the CCR metric, also has the smallest relative standard deviations (Table 3).

**Table 3:** Dependency on the χ cutoff value using models generated from datasets with 250 actives (*n*) on 10,000 compounds in total (*N*)

Metric	*χ*					*λ*
	0.5%	1%	2.5%	5%	10%	
PM	0.52 ± 0.25	0.57 ± 0.15	0.60 ± 0.08	0.60 ± 0.06	0.60 ± 0.04	1
ROCE	1.60 ± 1.19	1.59 ± 0.81	1.58 ± 0.51	1.55 ± 0.35	1.52 ± 0.23	
EF	1.54 ± 1.10	1.56 ± 0.76	1.55 ± 0.48	1.53 ± 0.33	1.50 ± 0.22	
REF	3.86 ± 2.75	3.89 ± 1.90	3.88 ± 1.20	7.63 ± 1.65	14.97 ± 2.22	
CCR	0.50 ± 0.00	0.50 ± 0.00	0.51 ± 0.01	0.51 ± 0.01	0.53 ± 0.01	
MCC	0.01 ± 0.01	0.01 ± 0.01	0.01 ± 0.01	0.02 ± 0.01	0.03 ± 0.01	
CKC	0.00 ± 0.01	0.01 ± 0.01	0.01 ± 0.01	0.02 ± 0.01	0.02 ± 0.01	
SEN	0.01 ± 0.01	0.02 ± 0.01	0.04 ± 0.01	0.08 ± 0.02	0.15 ± 0.02	
SPE	1.00 ± 0.00	0.99 ± 0.00	0.98 ± 0.00	0.95 ± 0.00	0.90 ± 0.00	
PRE	0.04 ± 0.03	0.04 ± 0.02	0.04 ± 0.01	0.04 ± 0.01	0.04 ± 0.01	
ACC	0.97 ± 0.00	0.97 ± 0.00	0.95 ± 0.00	0.93 ± 0.00	0.88 ± 0.00	
PM	0.96 ± 0.01	0.96 ± 0.01	0.96 ± 0.00	0.94 ± 0.00	0.91 ± 0.00	20
ROCE	28.80 ± 8.24	26.73 ± 5.20	22.13 ± 2.46	16.72 ± 1.11	10.49 ± 0.34	
EF	16.67 ± 2.70	16.12 ± 1.85	14.44 ± 1.03	11.99 ± 0.56	8.48 ± 0.22	
REF	41.68 ± 6.74	40.30 ± 4.62	36.09 ± 2.56	59.97 ± 2.79	84.78 ± 2.18	
CCR	0.54 ± 0.01	0.58 ± 0.01	0.67 ± 0.01	0.78 ± 0.01	0.88 ± 0.01	
MCC	0.18 ± 0.03	0.24 ± 0.03	0.34 ± 0.03	0.40 ± 0.02	0.40 ± 0.01	
CKC	0.13 ± 0.02	0.22 ± 0.03	0.34 ± 0.03	0.38 ± 0.02	0.31 ± 0.01	
SEN	0.08 ± 0.01	0.16 ± 0.02	0.36 ± 0.03	0.60 ± 0.03	0.85 ± 0.02	
SPE	1.00 ± 0.00	0.99 ± 0.00	0.98 ± 0.00	0.96 ± 0.00	0.92 ± 0.00	
PRE	0.42 ± 0.07	0.40 ± 0.05	0.36 ± 0.03	0.30 ± 0.01	0.21 ± 0.01	
ACC	0.97 ± 0.00	0.97 ± 0.00	0.97 ± 0.00	0.96 ± 0.00	0.92 ± 0.00	

The PM is not so much dependent on the applied cutoff value. For good models the EF and ROCE metrics decrease when the cutoff is increased, while the REF, CCR, MCC and CKC values always increase when the cutoff is increased from 2.5% up to 10%

### Dependency on both model quality *λ* and cutoff threshold *χ*

**Fig. 4:**
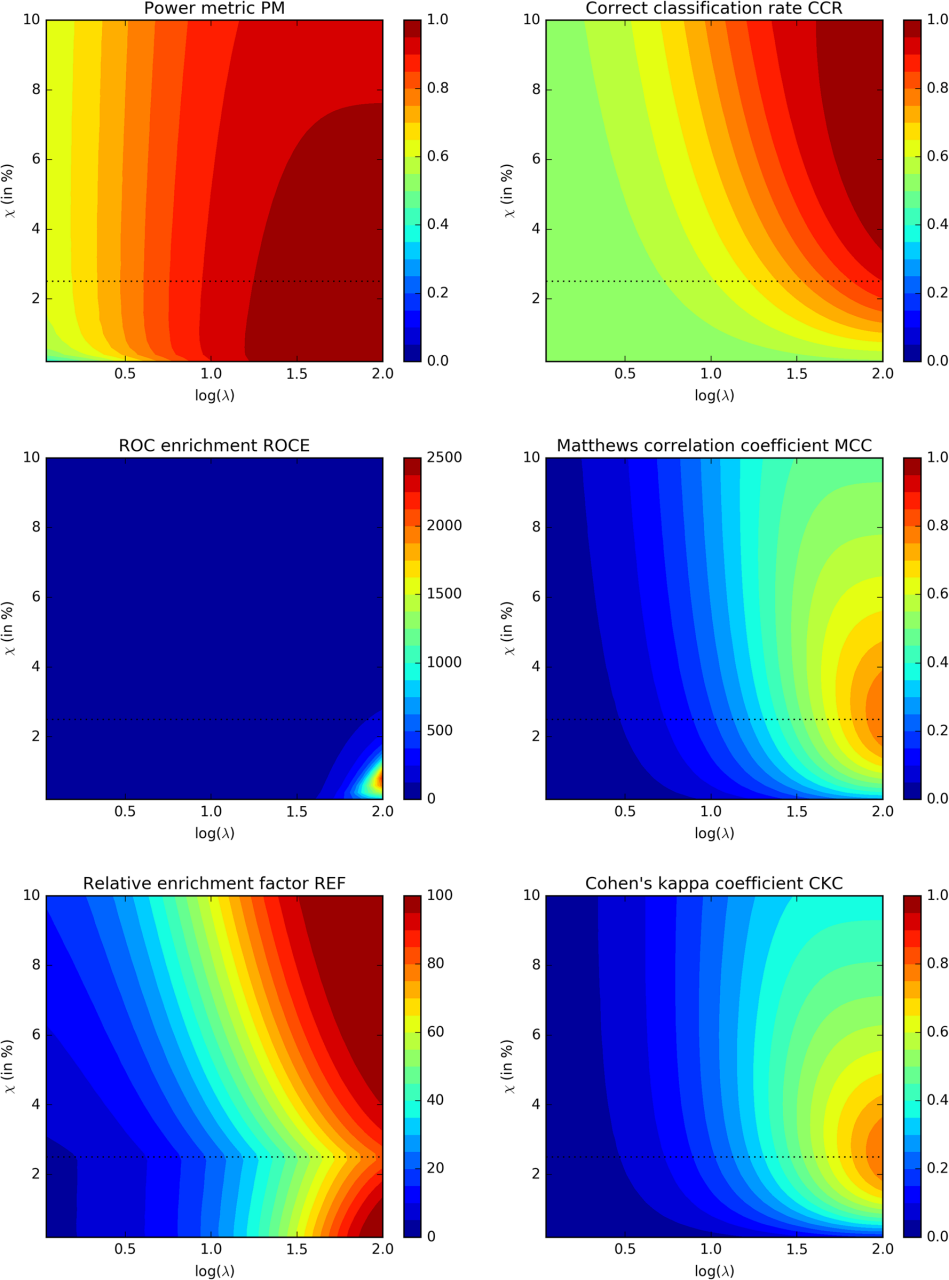
Comparison of the power metric with the five main other metrics (CCR, ROCE, MCC, REF and CKC) using a model dataset of 250 active compounds on a total number of 10,000. The logarithm of the quality parameter *λ* is varied along the abscissa [a log(*λ*) of 2 corresponds to a quality *λ* of 100] while the applied cutoff threshold *χ* is varied along the ordinates. The *black dotted line* at a cutoff value *χ* of 2.5% indicates the boundary of 250 compounds on a total of 10,000. In a perfect model, all 250 active compounds would be located along the topside of this boundary

A direct comparison of the variation of the values of the five most commonly used metrics (CCR, ROCE, MCC, REF and CKC) with those of the PM, as a function of both model quality *λ* and cutoff threshold *χ*, is provided in Fig. 4. Comparing the results of the PM and CCR metrics, both types of metric values increase with increasing model quality, but the PM metric seems to be less dependent on the applied cutoff threshold as compared to the CCR metric (in fact, the CCR metric value is increasing with increasing cutoff thresholds, while the opposite behavior is observed in the case of the PM metric). The CCR metric is finding its highest values at larger cutoff thresholds in combination with high model qualities, making it less suitable for early-recognition problems. A similar conclusion can be drawn for the MCC and CKC metrics, as in both cases maximum values are obtained near a cutoff threshold *χ* that is equal or close to the fraction of true actives within the entire dataset (in the example of Fig. 4, this is 2.5%). Focusing on the ROCE metric, maximum values are calculated when models of high qualities are combined with cutoff thresholds χ that are smaller than 2.5%, *in casu* the fraction of true actives within the entire dataset of compounds. At very low cutoff thresholds, the ROCE metric decreases again. A main disadvantage of the ROCE metric is the lack of a well-defined upper boundary, hence making it difficult to compare the quality of underlying models and applied cutoff thresholds. Finally, the REF metric is not a continuous function but shows a discontinuity in its metric value along a threshold cutoff value of 2.5%, a value that is equal to the fraction of true actives in the dataset. At this cutoff threshold value and for all model qualities, a minimum in metric value is observed, which makes that for any given model quality under consideration two maxima are found: a first optimum at a cutoff threshold smaller than the 2.5%, and a second optimum that is located at a cutoff threshold *χ* much larger than the 2.5%.

Based on these observations, it can be concluded that the CCR, MCC and CKC metrics are all less suitable for early-recognition problems; for these problems the PM and ROCE metrics are better suited. The REF metric might also be an option to some extend but some cautions are warranted when used in combination with cutoff thresholds *χ* that are equal or larger than the fraction of true actives in the entire dataset. In these cases an increase in the REF metric is observed, which makes it less suitable for early-recognition problems. As already mentioned, the main disadvantage of the ROCE metric is the lack of a well-defined upper boundary, and for this reason the PM metric seems to posses powerful early-recognition properties and might be one of the preferred metrics for evaluating virtual screening models.

## Conclusions

The power metric PM as described in this paper is a statistically solid metric with little sensitivity to the ratio of actives to the total number of compounds (the *R*_*a*_ value; see Table 2) and little sensitivity to the cutoff threshold parameter χ (Table 3). The metric is dependent on the underlying model quality, in such sense PM values around 0.5 are calculated for poor to random models, and values between 0.9 and 1.0 for high quality models. It is statistically robust in the sense that the calculated standard deviations are small and largely insensitive to the applied threshold cutoff value χ.

## Abbreviations

ACC: accuracy
AUC: area under the curve
CCR: correct classification rate
CKC: Cohen’s Kappa coefficient
EF: enrichment factor
MCC: Matthews correlation coefficient
PM: power metric
PRE: precision
QSAR: quantitative structure–activity relationship
REF: relative enrichment factor
ROC: receiver operating characteristic
ROCE: ROC enrichment
SEN: sensitivity
SPE: specificity
STD: standard deviation
TNR: true negative rate
TPR: true positive rate
